# Automated multimodal fluorescence microscopy for hyperplex spatial-proteomics: Coupling microfluidic-based immunofluorescence to high resolution, high sensitivity, three-dimensional analysis of histological slides

**DOI:** 10.3389/fonc.2022.960734

**Published:** 2022-10-13

**Authors:** Laura Furia, Simone Pelicci, Federica Perillo, Maddalena M. Bolognesi, Pier Giuseppe Pelicci, Federica Facciotti, Giorgio Cattoretti, Mario Faretta

**Affiliations:** ^1^ Department of Experimental Oncology, European Institute of Oncology IRCCS, Milan, Italy; ^2^ Department of Oncology and Hemato-Oncology, University of Milan, Milan, Italy; ^3^ Department of Medicine and Surgery, Università di Milano-Bicocca, Monza, Italy; ^4^ Department of Biotechnology and Biosciences, University of Milan-Bicocca, Milan, Italy

**Keywords:** multiplexing, fluorescence microscopy, automation, microfluidics, image analysis

## Abstract

*In situ* multiplexing analysis and *in situ* transcriptomics are now providing revolutionary tools to achieve the comprehension of the molecular basis of cancer and to progress towards personalized medicine to fight the disease. The complexity of these tasks requires a continuous interplay among different technologies during all the phases of the experimental procedures. New tools are thus needed and their characterization in terms of performances and limits is mandatory to reach the best resolution and sensitivity. We propose here a new experimental pipeline to obtain an optimized costs-to-benefits ratio thanks to the alternate employment of automated and manual procedures during all the phases of a multiplexing experiment from sample preparation to image collection and analysis. A comparison between ultra-fast and automated immunofluorescence staining and standard staining protocols has been carried out to compare the performances in terms of antigen saturation, background, signal-to-noise ratio and total duration. We then developed specific computational tools to collect data by automated analysis-driven fluorescence microscopy. Computer assisted selection of targeted areas with variable magnification and resolution allows employing confocal microscopy for a 3D high resolution analysis. Spatial resolution and sensitivity were thus maximized in a framework where the amount of stored data and the total requested time for the procedure were optimized and reduced with respect to a standard experimental approach.

## 1 Introduction

Heterogeneity is a hallmark of cancer challenging the comprehension of its molecular basis and the definition of successful therapies (1–8). Its origin can be attributed not only to the clonal evolution of a genetic diversity among the cells in the tumor, but it can also be referred to the network of interactions established among cancer cells and the host environment (8, 9). Knowledge of the Tumor Micro Environment (TME) is now considered an important and integrating factor in understanding cancer origin, growth and potential response to therapy (10–12). Profiling of the genetic traits of cancer and definition of the resulting transcriptional programs are nowadays efficiently performed by Next Generation Sequencing techniques. However, to take into consideration the heterogeneous nature of the disease, it is absolutely necessary to progress towards single-cell resolution analysis (13). Unfortunately, NGS technologies loose the enormous amount of information that resides in the analysis of tumor architecture in-situ and in the description of the surrounding host environment with the cell-cell interactions residing therein.

As a result, a dramatic effort in the creation of imaging tools to support the so called “spatial biology” has been started. Microscopy can serve as a guide to localize small areas to be physically removed from a tissue sample and sequenced by NGS (14–17).

On the other hand, both spatial transcriptomics and in-situ cell immunophenotyping progressed till reaching a content of several thousands of transcripts and a hundred of proteins (17–25).

Sequential immunostaining protocols (26–30), based on the repetition of staining/image-collection steps intercalated by removal of the bound antibodies, provided a first approach to multiplexed immunophenotyping. Next to these ones, methods based on DNA oligo-conjugated antibodies, detected by sequential in-situ hybridizations towards selected tagging primers, opened an alternative way to an easier removal of the bound fluorochromes (26, 31, 32).

Even if these works contributed to make single-cell multi-omics more and more available for scientists, a big effort is requested to enhance the applicability of these techniques to the routine of the research laboratory and clinical histopathology.

The high complexity of a multiplexing analysis targeted at the detection of either RNA transcripts or proteins imposes several challenges to create standard workflows to be employed into the clinical routine. Process automation is instrumental for performing the complex tasks required by multiplexing analysis with the goal i) to provide repeatability, ii) to standardize sample preparation protocols and iii) to reduce the subjectivity related to human intervention. All the phases from sample preparation to image collection and data analysis are suitable to be automatized. Completely robotized workflows can thus generate data to be finally analyzed by researchers and physicians.

Sample preparation can benefit of the progress made by miniaturization giving rise to a continuously growing number of lab-on-chip devices to control a wide variety of operations including immunostaining and *in-situ* fluorescence hybridization (33–37).

Moreover, automation made possible to combine staining and imaging processes with the creation of a novel class of instruments able to perform all the steps of a multiplexing protocol. Various companies now offer slide- stainer/scanner, to process single or multiple samples, where the imaging apparatus is incorporated into a staining device.

Slide scanners are nowadays the most common imaging solution employed for multiplexing analysis. They offer the advantage of an optimized acquisition time for large tissue samples granting single cell resolution and robust data collection for subsequent cell segmentation and fluorescence analysis.

Even if fluorescence microscopes possess all the features to efficiently perform the same tasks, they are not widely diffused as detection tools in high-plex histopathology. Acquisition by fluorescence scanners grants an easier approach and a higher sample throughput by dedicated acquisition tools specifically developed for the histopathological routine (38). However, the vast majority of scanners performs data acquisition by dry objectives limiting the available numerical aperture (NA) to values less than 1. It is worthwhile to remind that increasing NA, by using immersion objectives, not only provides higher spatial resolution but also dramatically enhances signal sensitivity. Thus analysis of the results can be greatly facilitated by the high signal-to-noise ratio in particular when low expressed antigens were targeted. More than this, performing experiments of *in-situ* transcriptomics to detect single RNA molecule (smRNA) by FISH requires high sensitivity and high spatial resolution to precisely measure the amount of targeted transcripts. Finally, the modern fluorescence microscope is the core for a wide range of technologies ranging from confocal to super resolution microscopy allowing, for example, to perform optical sectioning analysis in order to get a real 3D spatial distribution of the molecules in the tissue.

We present here an experimental pipeline based on alternate manual and automated sample immunostaining by a commercial microfluidic high-pressure stainer. Staining automation allowed a dramatic reduction in the duration of multiplexing experiments. A sequential immunostaining procedure based on the MILAN protocol (30) can detect several different antigens located in various cellular compartments with an optimized signal and with a dramatic cut in the total duration of the experiment. We thus characterized the performances of the fast high-pressure stainer on targeted reference antigens in terms of saturation with respect to the standard manual staining protocols.

In order to provide the best balance between employed time, resources and results quality we developed a data collection procedure based on a variable resolution acquisition performed by a motorized fluorescence microscope. We completed the pipeline by a series of open-source computational tools to register the different acquisition steps thus producing the final multiplexed images.

We finally exploited the potential of a variable optical resolution by acquiring diffraction limited images and combining them with optically sectioned 3D stacks demonstrating that the alternate use of automated and manual sample preparation procedures can optimize the final results of a multiplexing analysis by introducing high resolution information and thus increasing the final content of the analysis.

## 2 Materials and methods

### 2.1 Formalin-fixed paraffin-embedded samples

A Tumor Micro-Array of 96 cores (diameter of 1.2mm) of FFPE human ColoRectal Cancer specimens were obtained from San Gerardo Hospital in Monza. Sections of 3 ± 1 μm thickness were placed on positively charged standard microscope slides (SuperFrostRPlus; Bio-Optica, Milan, Italy). The study has been approved by the Institutional Review Board Comitato Etico Brianza, N. 3204, “High-dimensional single cell classification of pathology (HDSSCP)”, October 2019. Patients consent was obtained or waived according to article 89 of the EU general data protection regulation 2016/679 (GDPR) and decree N. 515, 12/19/2018 of the Italian Privacy Authority. The study is a retrospective study and no clinical trials apply.

### 2.2 Multiplexing staining (MILAN protocol)

Slides at the bench were stained according to the previously published protocol (30). After every acquisition, slides underwent stripping to completely remove bound antibodies. The stripping buffer (SDS 10% (Sigma-aldrich, St. Louis, MO, USA), Tris HCl 0,5M pH6,8 (Sigma-aldrich, St. Louis, MO, USA)) was preheated to exactly 56°C in closed, shaking water-bath. Once the solution reached the temperature, β-mercaptoethanol 0,4% (Sigma-aldrich, St. Louis, MO, USA) was added and the slides were inserted into a glass container for 30 minutes. After stripping, slides were subjected to several Tris-Buffered Saline – Tween20 sucrose (TBS-Ts) (Sigma-aldrich, St. Louis, MO, USA) pH 7.5 1X solution washing steps before being restained.

### 2.3 Automated staining

Automated staining was performed by a LabSat microfluidic stainer (Lunaphore Technologies SA, Tolochenaz, Switzerland). The staining protocol was compiled with the LabSat Development Software. Incubation time of antibodies was varied to test staining efficiency as explained in the text. Antigen Retrieval and stripping were performed according to the manufacturer instructions.

### 2.4 Antibodies

Primary Abs used for multiplexing (**Table 1**) were diluted to the specific final concentration with Abs diluent (TBS, 2% BSA, 0.05% Sodium Azide and 100 mM Trehalose (Sigma-aldrich, St. Louis, MO, USA)) and incubated Overnight. Secondary Abs (**Table 2**), diluted in PBS 1X, were added after washing steps in TBS-Ts and PBS 1X. Secondary antibodies were incubated for 30 minutes at room temperature in a dark humid chamber. After 3 washing steps, tissues were washed and then stained with DAPI (2,5 μg/ml, Sigma-aldrich, St. Louis, MO, USA) in the dark for 10 minutes. The coverslip was mounted using Prolong mounting media (ThermoFisher Scientific, Waltham, MA, USA).

**Table 1:** List of the employed primary antibodies.

Name	Marker for (cellular localization)	Isotype	Cat n.	Source	Concentration
Anti-CD3	T cell (Surface)	Rabbit	A0452	Dako	6 µg/ml
Anti-Lamin A	Nuclear Marker	Mouse IgG3	MA1-06101	Thermofisher	10 µg/ml
Anti- Ki67 Alexa647	Proliferating cells (Nuclear)	Mouse IgG1	558615	BD-Bioscience	5 µg/ml
Anti- Ki67	Proliferating cells (Nuclear)	Mouse IgG1	550609	BD-Bioscience	5 µg/ml
Cytokeratin8	Epithelium	Mouse IgG2a	sc-58736	Santa-Cruz Biotechnologies	5 µg/ml
γH2A.X	DNA Damage Response	Mouse IgG1	613402	Biolegend	5 µg/ml
53BP1	DNA Damage Response	Rabbit	36823	Abcam	5 µg/ml

**Table 2:** List of the employed Secondary Antibodies.

Reagent	Cat n.	Source	Concentration
Alexa Fluor^®^ 488 AffiniPure Donkey Anti-Rabbit IgG	715-545-152	Jackson Immunoresearch	7,5 µg/ml
Alexa Fluor^®^ 790 AffiniPure Goat Anti-Mouse IgG, (IgG3)	115-655-166	Jackson Immunoresearch	8,5 µg/ml
Alexa Fluor^®^ 647 AffiniPure Goat Anti-Mouse IgG, (IgG2A)	115-607-186	Jackson Immunoresearch	8,5µg/ml
Alexa Fluor^®^ 488 AffiniPure Goat Anti-Mouse IgG, (IgG1)	115-545-205	Jackson Immunoresearch	8 µg/ml
Alexa Fluor^®^ 647 AffiniPure Goat Anti-Mouse IgG, (IgG1)	115-605-205	Jackson Immunoresearch	8,5 µg/ml
Cy3 AffiniPure Donkey Anti-Rabbit IgG	711-165-152	Jackson Immunoresearch	8,5 µg/ml
Alexa Fluor^®^ 488 AffiniPure Goat Anti-Mouse IgG, (IgG2A)	115-545-206	Jackson Immunoresearch	7,5 µg/ml

### 2.5 smRNA fluorescence *in-situ* hybridization

To detect RNA transcripts, slides were stripped after the last immunostaining and subjected to several TBS-Ts washing steps before starting the RNA-FISH staining procedure. The two employed probes were synthetized to detect Actin B1 and B-RAF mature RNA transcripts (Molecular Instruments Inc., Los Angeles, CA, USA). According to the manufacturer protocol (HCR RNA-FISH for FFPE Tissue Sections), slides were incubated with hybridization buffer in humidified chamber for 10 minutes at 37°C and then incubated overnight with probe solution containing 0.4pmol of each probe mixture in humidified chamber at 37°C. The day after, slides were rapidly washed with 30% probe wash buffer and subsequently with buffers containing different percentages of probe wash buffer + 5 X SSCT (75% + 25%, 50% + 50%, 25% + 75%) for 15 minutes each one at 37°C. The last wash was performed in 100% 5X SSCT for 15 minutes at 37°C. Slides were finally incubated with amplification buffer in humidified chamber for 30 minutes and incubated with hairpin solution containing 6pmol of each hairpin fluorescent probe overnight in a dark humidified chamber at room temperature. Samples were then washed several times with 5 X SSCT at room temperature and mounted as specified above.

### 2.6 Image acquisition

#### 2.6.1 Microscopes set up

An inverted Nikon Eclipse Ti2 microscope (Nikon instruments, Tokyo, Japan), equipped with a LED light source (pE-4000 CoolLED, Andover, United Kingdom), was used to acquire multicolor widefield fluorescence images. Emitted light was collected by a CMOS camera (Dual ORCA Flash 4.0 Digital CMOS camera C13440, Hamamatsu, Japan) set on a 16-bit scale detection modality. Optimal exposure time was set per each fluorescence channel by maximizing the dynamic range and avoiding saturation based on a preliminary observation of randomly chosen cores (20x: from 80 to 400 ms; 60x: from 50 to 200 ms). Slides were stained with DAPI to visualize nuclei, and secondary antibodies conjugated with three different fluorophores to visualize all the antigens of interest: Alexa Fluor 488, Cy3 and Alexa Fluor 647 (**Table 2**). In the experiments performed to compare automated and manual staining efficiency an Alexa Fluor 790 secondary antibody was employed to minimize background thanks to the complete absence of autofluorescence in this spectral range. Single cell resolution images were acquired by a 20x Plan Apo 0.75 NA objective, while high resolution imaging was performed by a 60x Plan Apo 1.4 NA objective.

The microscope is equipped with an A1R confocal scanhead to acquire optically-sectioned multiplane stacks. The excitation laser bench is composed of the following excitation lines: 405 nm (23.1 milliWatts), 488 nm (79.1 milliWatts), 561 nm (79 milliWatts), 647 nm (137 milliWatts). All the indicated powers were measured at the exit of the excitation optical fiber. Acquisitions were sequentially performed per each fluorophore with the high-speed galvanometric mirrors at a speed of 15 frames per second (2x Average) to minimize photobleaching and collection time. The slit aperture of the spectral detection system was set to maintain an optimal Signal-to-Noise ratio avoiding crosstalk among the different channels (namely 500-530 nm for the green channel, 570-600 nm for the orange one and 660-720 nm for the far red). Pinhole size has been set to 1.0 Airy Unit for every collected channel.

### 2.7 Data acquisition protocol

Data acquisition was managed by the NIS Elements software version 5.30.07 (Nikon instruments, Tokyo, Japan). TMA and cores are chosen as demonstration: the detailed procedure below also applies to a generic sample with regions defined according to more general criteria. The acquisition protocol was coded into a routine of the Microscope Control Software (see **Supplementary Material**: multiplexingcenterTMA.bin) and is composed of different steps:

1. The entire slide was first acquired by a 4×/N.A. 0.13, and the resulting image (from now on named Map) used to locate the Regions Of Interest (ROIs).
2. Single cores were segmented either manually or automatically by a macro written for the ImageJ software (W. Rasband, National Institute of Health, USA). The corresponding masks with the positions of the ROIs was then stored and opened by the microscope control software to set the stage coordinates for the next image collection.
3. Cores were re-located and images acquired as the result of a multipoint acquisition, with 10% overlap among consecutive images. Image stitching was then executed in order to reconstruct the entire field of interest. A square field of view of 1.4 mm was chosen to ensure imaging of the entire tissue spot. When working at single cell resolution with a 20x 0.75 NA dry objective, a hardware-based focus control (Perfect Focus System (PFS), Nikon instruments, Tokyo, Japan) was employed. Optimal offset was estimated core-by-core by an image autofocus procedure applied to the DAPI channel.
4. In multiplexing experiments, the stripped and re-stained slice was repositioned on the stage and reacquired at low resolution as indicated at point 1.
5. Image acquisition parameters were first re-optimized before launching the next acquisition routine of the control software.
6. The acquired low resolution map was aligned to the one collected in the previous acquisitions employing an ad-hoc registration routine implemented in ImageJ software (**Supplementary Material**: 1.8 MacroRegTwoImagesforNIS_22072022). The calculated parameters of the roto-translation required to correct the spatial shift among the different acquisitions were then automatically applied to the stored ROIs and passed to the microscope control software (see Image Analysis Section).
7. ROIs were reacquired with the new imaging modalities. For high resolution widefield imaging a 60x N.A. 1.4 Oil-Immersion objective was employed. To maintain the optimal focal plane over the entire region, an autofocus map was built by running an autofocus algorithm at different points and the resulting values were then interpolated to calculate the coordinates of the plane of focus.
8. The same procedure was applied to recalculate the position of the ROIs to be imaged by confocal imaging.

### 2.8 Image analysis (AMICO analysis package)

The acquired images were processed by a series of newly developed computational tools based on the Automated Microscopy for Image-Cytometry (A.M.I.CO.) analysis package (39–41) adapted to multiplexed histological imaging. The software has been modified to process the multimodal multiresolution data created in the present work. The software is freely available upon request or available on GitHub public repository (https://github.com/MarioFaretta/AMICO). Since it is not possible to code to all the information required (e.g. format of the position lists for the microscope, format of the acquired images and metadata) by different microscope brands, a customization step is required to the users for adapting the code to their set-ups. We are available to provide help for these modifications.

#### 2.8.1 Data preparation and image registration

Image registration was performed by an ImageJ-macro code based on the TurboReg registration plugin present in ImageJ (https://github.com/MarioFaretta/AMICO). For full-size large images, registration algorithms were applied to the downscaled data (e.g. 50%, 25%) and then recalculated for the original size before file saving. Once concluded the registration of DAPI stained images, the calculated transformation parameters (i.e. angle of rotation and distance of translation) were then applied to the ROI mask and stored in a dedicated directory.

The same procedure was applied to the acquired single-cell resolution and diffraction-limited images before image cytometry analysis: ROIs registration at low resolution (4x) can sometime lead to minor shifts between images taken at higher magnification (20x, 60x) requiring a fine correction of the spatial shift. In this case the transformation parameters were calculated on the DAPI signal and then applied to all the fluorescence channels. To register images from different magnification, they were first rescaled to the same pixel dimension and then registered as specified.

#### 2.8.2 Image-cytometry analysis

The Image-Cytometry analysis core of the A.M.I.CO platform was then employed to proceed in the TMA analysis. The package is described in detail in a previous work (39, 40).

• Briefly, a first module (A.M.I.CO_Union) executes the image analysis steps required for the recognition, localization and measurement of single cells in the tissue sample/core. DAPI-based segmentation, with ad-hoc spatial-clusters decomposition, was performed. A novel routine was developed to delineate single cell borders according to the Cytokeratine signal or Membrane Markers (e.g. CD4, CD8). Image analysis was then executed in batch for all the selected cores in the acquired TMA. Single cell measurements were organized into a database containing in each row: cell identity (in the form of a progressive number used as a tag), image localization, physical size (Cell Area, calculated as number of pixels contained in the segmented-cell mask) and geometry (Circularity: cell shape descriptor calculated as 4π*Area/(Perimeter)^2 according to the ImageJ definition. It takes value 1 for perfect circles. It approaches 0 for increasingly elongated cells), single cell fluorescence measurements for every channel (Total Intensity: Sum of the intensity values calculated over the pixels contained in the segmented cell mask; Mean Intensity: Mean Intensity value of the pixels contained in the segmented cell mask) and sub-compartments related measurements (Number of spots/structures per cell; Total Intensity of the spots/structures: Sum of the intensity values calculated over the pixels of all the segmented spot/structure masks contained in a cell; Mean intensity per spot/structure: Average of the Mean Intensity values of the spots/structures contained in a cell, calculated as Mean Intensity value of the pixels in each segmented spot/structure mask).

A data analysis module (A.M.I.CO_Plotting) was employed to perform image-cytometry analysis to calculate single-cells statistics according to a classical flow-cytometry interface. Histograms and 2D multicolor dot-plots were generated to represents all the events in the sample allowing the definition of specific regions for statistical measurements and logical gates for selective targeting of events. Physical cell-location (baricentrum cell coordinates) could also be retrieved to select subpopulations of interest. The software can now create images containing the masks of the targeted populations: the defined regions can be re-used to perform additional analysis and/or to locate events to be acquired with different imaging modalities as explained above.

#### 2.8.3 Image-cytometry analysis of the automated-staining conditions

To compare results from the different incubation times employed for the automated stainings, images of different areas within the tissue samples were segmented by the DNA signal. CD3 images were then selected to ensure the presence of positive cells. Analysis of the CD3 fluorescence was also based on the possibility to clearly identify a positive-cell population. However, it was not possible to consider the calculated percentage as a parameter for direct comparison due to the heterogeneity in the number of positive cells in the employed samples. In all the analysis the mean intensity per pixel per cell was employed as a reference for the comparison. The reported images were converted to an 8-bit representation from the original 16-bit scale maintaining a fixed intensity scaling to allow visual comparison.

## 3 Results

### 3.1 Evaluation of the efficiency of automated immune-staining by a high-pressure microfluidic tissue processor

We previously published a protocol for multiple iterative labelling of tissue sections based on immunostaining by routinely employed antibodies followed by sequential stripping and restaining (30). The protocol was then inserted into an image collection and analysis pipeline for tissue cytometry.

To reduce the total duration of a single multiplexing experiment and to increase reproducibility, we introduced in the pipeline the automation of the sample staining by a commercially available tissue microprocessor. The employed system is based on a microfluidic chip capable of efficient delivery of the reagents on the tissue slide thanks to an applied high pressure that allows reducing the staining time to minutes (34).

Some preliminary tests (**Supplementary Figure 1**; **Supplementary Table 1**) revealed that the automated staining process allowed a dramatic decrease in the total sample-preparation time (**Supplementary Table 2**). Even if the staining patterns were in agreement with the expected ones, the signal-to-noise ratio greatly varied from antigen to antigen when compared to the standard manual-staining conditions (**Supplementary Figure 2**).

We thus decided to analyze the staining performances of the tissue processor in comparison with the standard overnight incubation adopted in the MILAN protocol (30). A slice of colon tissue was stained with different antibodies. CD3 was chosen as a representative membrane marker to test the staining efficiency of easily accessible and highly expressed antigens. Nuclear lamin A was measured instead as a representative target for the nuclear compartment. Finally, a directly conjugated antibody against the KI67 proliferation marker was selected to verify the results of the automated fast staining conditions in absence of signal amplification. The incubation time was varied to find the minimum duration required for antigen detection with a signal-to-noise ratio able to grant reliable results.

**Figure 1:**
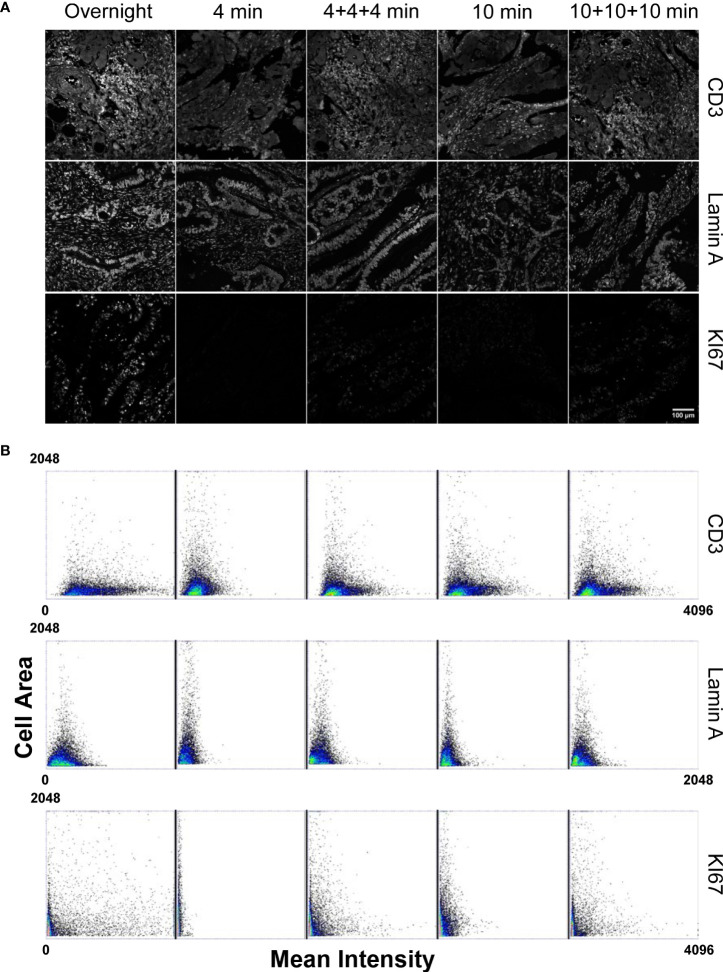
Comparison of the staining performances of a microfluidic based automated stainer at different incubation timing versus manual overnight procedure. (Panel **A**) Staining conditions have been applied to: CD3 marker as representative of highly expressed and accessible antigens; Lamin A for nuclear, high expressed localization; KI67 directly conjugated antibody to test primary antibody staining efficiency without amplification. Scale bar: 100μm. (Panel **B**) Dot Plots, obtained from single cell measurements (every spot in the graph represents a single cell), reporting the Mean Intensity of CD3, Lamin A and KI67 in relation to the single cell dimension (Cell Area) for different incubation times. Mean Intensity was calculated as the mean value of the pixels contained in the segmented cell mask. Cell area corresponds to the number of pixels contained in the segmented cell mask.

Analysis of the CD3 antibody response (**Figure 1**) revealed that, even if antigen saturation has not been reached in the fast staining conditions employed by the tissue microprocessor, the resulting data provided reproducible measurements. As incubation time was reduced, the registered mean intensity decreased with respect to a standard overnight incubation performed at the bench as demonstrated by the contraction of the events distribution in the dot plots. However, it has to be considered that membrane markers are commonly employed to identify immune cells by placing an intensity threshold to define a positive cell population. CD3 positive cells can be efficiently detected in all the tested cases. The low signal to noise ratio obtained with the shortest incubation (4 min) made the threshold determination harder but the other adopted conditions produced very similar distribution that clearly demonstrated the efficiency of the procedure.

Detection of a nuclear marker was generally aimed at a precise quantification of the amount of protein expressed in single cells in a tissue. We thus measured mean intensity of Lamin A as a read-out of molecular density per cell under different incubation times. The dot plots in **Figure 1** evidenced how the signal reached after an overnight incubation cannot be reproduced by the tissue processor in any conditions. A progressive reduction of the extension of the intensity distribution as incubation time decreased was instead observed. Repeated injections of fresh antibodies and/or prolonged exposure times did not lead to significant variations in the detected response with respect to the values detected after overnight incubation at the bench demonstrating that the price to pay for the dramatic cut in the duration of the experiment was a diminished sensitivity in signal quantification.

Analysis of another nuclear marker, the proliferation-related antigen KI67 that is abundantly expressed and well detected by secondary antibody amplification, revealed that the reduction in the generated intensity was essentially due to a reduced amount of primary antibody bound to the target. Measurements of the intensity generated by the directly conjugate antibody (**Figure 1** panel B, KI67 dot plots) showed a dramatic dependence from the adopted incubation conditions.

For all the examined cases, repeated injections of fresh reagents contributed more efficiently to a partial recovery of the measured signals.

In summary, highly accessible and represented antigens are efficiently detected and measured even in the absence of signal saturation providing a dramatic reduction in time for the duration of a multiplexing experiment as reported in **Supplementary Table 1** and maintaining the conditions to set a robust positivity threshold. When a precise measurement of the total amount of expressed protein is required, switching to a manual staining procedure is suggested instead, in order to benefit of antigen saturation and of maximization of the signal to noise ratio.

### 3.2 Application of the multiplexed pipeline with variable resolution image collection: Combined immuno-multiplexed smRNA FISH analysis

**Figure 2:**
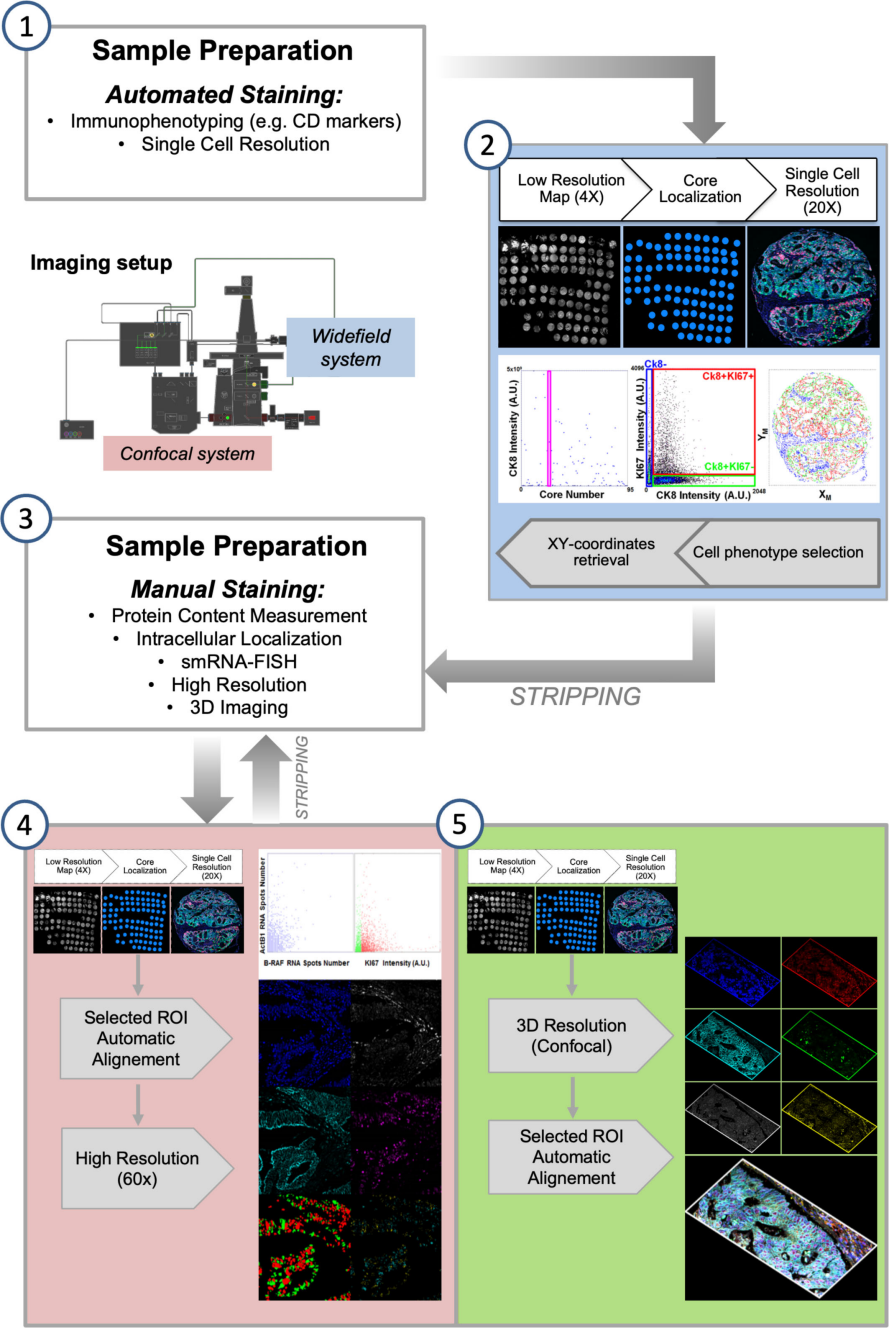
Semi-automated Multi-Modal Multi-Resolution Workflow for Multiplexing Experiments. (1) Samples are processed with an automated stainer for detection of highly expressed population-marking antigens. (2) Images are collected by an automated fluorescence microscopy workstation following an analysis-driven protocol that allows automatic selection and acquisition and processing of single cell-resolution data. Steps (1-2) can be iterated for some cycles by stripping bound antibodies. (3) Immuno-staining is performed at the bench to allow high sensitivity and resolution detection of selected biomarkers. (4) Diffraction limited and (5) 3D confocal analysis is performed on targeted regions calculated from the analysis of the cell-resolved data from step (2). Steps (4-5) can be iterated for some cycles by stripping bound antibodies.

Alternating automated and manual processing of the sample requires a compatible image collection step. One of the major computational efforts in the analysis of an iterative staining-stripping sequence is the registration of images acquired after every staining. Combined staining-imaging devices remove this obstacle by avoiding sample movements. Their use has been proposed to replace traditional scanners and fluorescence microscopes as acquisition tools for multiplexing. However, efficient image re-alignment can also grant single cell relocation over several acquired images allowing the successful employment of traditional detection systems (28, 30). In our previous works, we entirely performed the alignment procedure in the post-acquisition processing phase. We now developed a modification in the pipeline by inserting a first registration step during image collection. The acquisition workflow (**Figure 2**) starts with the fast construction of a low magnification map to target the region of interest (ROI) for the successive acquisitions at increased resolution. The ROI can coincide with the entire tissue slice(s), or can be confined to a specific histological area in the sample or e.g. can identify the cores of a Tissue Micro Array (TMA). After the first round of data collection, once re-acquired the low resolution map, the high resolution targeting ROIs were automatically repositioned according to the actual stage coordinates, in order to minimize the shift between different images and the computational times required for the post-acquisition final alignment.

Images taken with different magnification are registered to identify single cells: whole-cell resolution data (total amount of expressed markers, cell marker positivity) can be merged with highly resolved ones (number and intensity of intracellular structures, e.g. foci) calculated at full-resolution and reassigned to the targeted cell.

**Figure 3:**
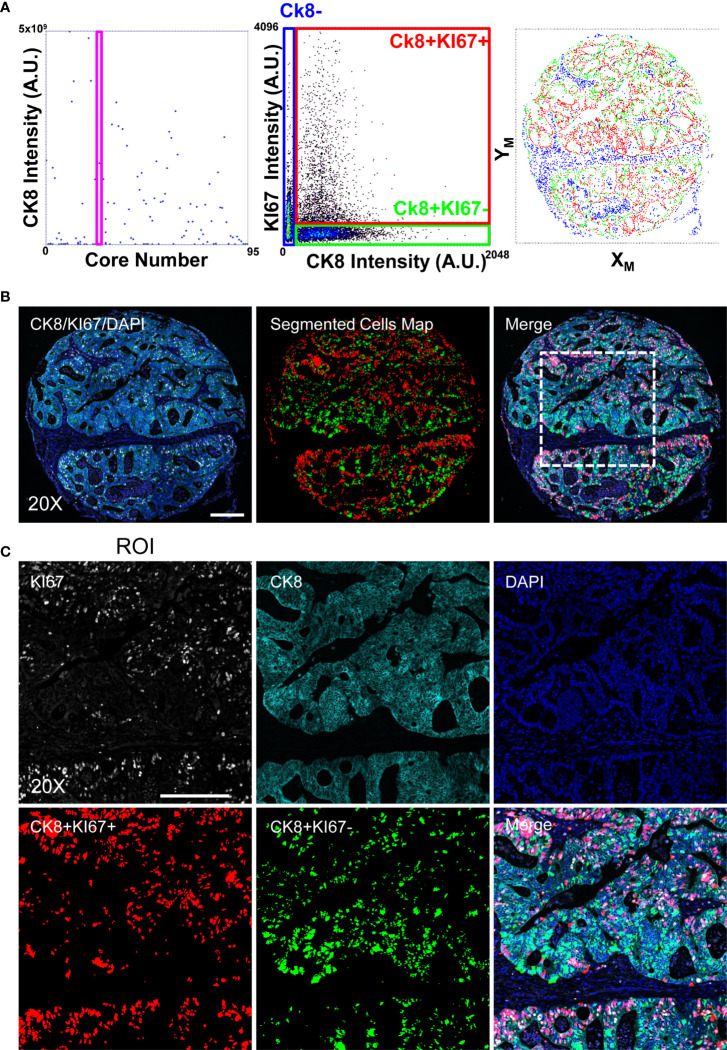
Image-Cytometry Analysis Workflow on a TMA. Single cell resolved images of single cores, collected according to the discussed protocol, are segmented and statistics of single cores calculated to select targets enriched in the epithelial regions (CK8+). Panel **(A)**: Targeted Cores (the shown representative core is identified by the Magenta Rectangle graph on the left) are then reanalyzed by segmenting cells in the CK8 positive area, reporting the expression of the KI67 proliferation marker (middle graph) to define the population of interest, i.e. proliferating (CK8+KI67+) and quiescent (CK8+KI67-) epithelial cells. Image coordinates of the cells (right graph) are then re-converted into microscope stage positions for the next acquisition. Panel **(B)**: 20X single cell resolved stitched image of the targeted core. Acquired channels (left), segmented cells (middle; CK8+KI67+(Red) and CK8+KI67-(Green)) and the resulting merge (right) are shown. The highlighted square identifies the ROI magnified in C. Scale bar: 200μm. Panel **(C)**: Enlarged views at full resolution of the KI67, CK8, DAPI fluorescence channels, together with segmented masks of the CK8+KI67+(Red) and CK8+KI67-(Green) cell populations. Scale bar: 200μm.

To validate the performances of the adopted approach, we analyzed a Tissue Micro Array composed of 96 cores from ColoRectal Cancer (CRC) biopsies. The sample was initially stained to detect the KI67 marker and Cytokeratin 8 (CK8) (**Figure 3**). Cores were classified according to the presence of epithelial areas: single cells were segmented employing CK8 signal as cell body delimiter and proliferative index was measured according their KI67 expression.

After antibody removal, the same TMA was stained to evaluate DNA damage by measuring levels and intracellular distribution of phosphorylated serine 39 of histone H2A.X (γH2A.X) and 53BP1 protein. Accumulation of γH2A.X and 53BP1 in foci, that represents the first step in the initiation of the canonical DNA Damage Response (DDR), localizes DNA Double Strand Breaks (DSBs) in cells providing a direct measurement of the genome integrity. While proliferation activity and DNA replication can be assessed by a labelling index calculated at cellular resolution, the recognition and counting of DSBs require high sensitivity and diffraction limited analysis in order to detect up to the smallest and dimmest foci to recapitulate the wide range of detectable phenotypes associated to DDR activation.

According to the previously performed analysis with 20x magnification, the KI67 labeling index in the epithelium (CK8+ regions) was measured (**Figure 3**), and cores containing large epithelial areas with high proliferative activity were selected. Then, after image registration, we evaluated the number and intensity of γH2A.X foci at the maximum resolution to obtain a measure of the DNA damage in regions with the potentially highest replication stress.

**Figure 4:**
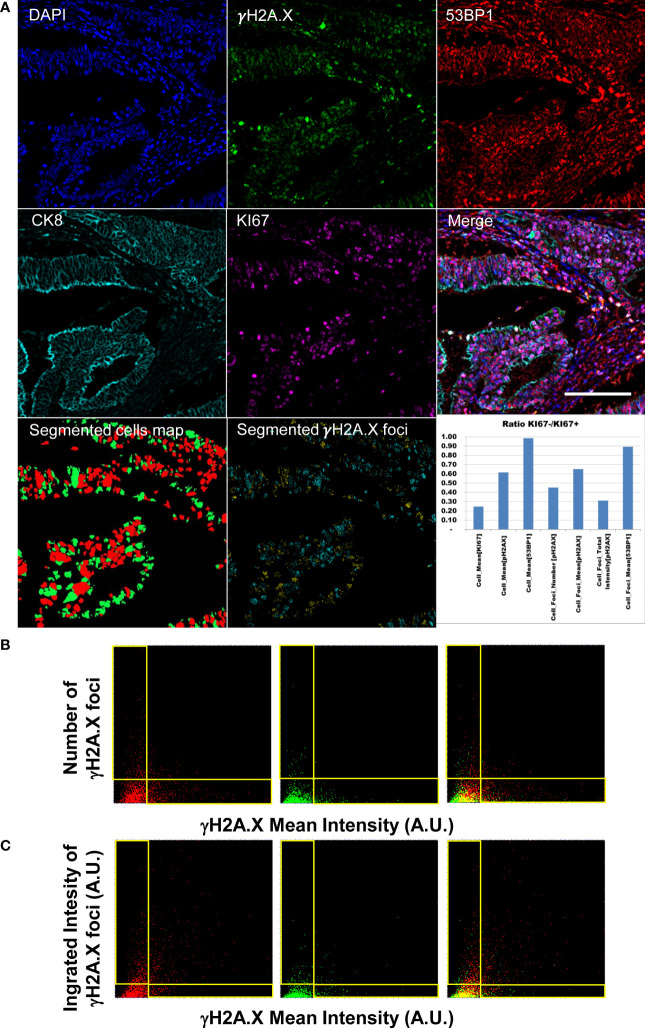
Diffraction-limited Image-Cytometry Analyis of a relocalized targeted core. Panel **(A)**: Images are collected with a 60x 1.4 Oil Immersion Objective to measure DDR related parameters at maximum spatial resolution and sensitivity on a selected region inside a previously acquired core: DAPI, γH2A.X, 53BP1, CK8 and KI67 (CK8 and KI67 rescaled to the same digital resolution from the previously acquired 20x acquisition employed to target the selected region; scale bar: 200μm). Segmented masks of the cells (KI67+, Red; Ki67- Green) and of γH2A.X foci of the isolated cell populations (KI67+ Cyan spots; KI67- Yellow spots) are also reported. The graph shows the ratio of DDR related parameters values calculated for the quiescent and proliferant cell population. Panel **(B)**: Dot Plots reporting single-cell DDR related parameters. Mean Intensity (calculated as Mean Intensity value of the pixels contained in the segmented cell mask) of cell nuclei do not correlate with the number of detected foci per cell as demonstrated by the L-shaped distribution (events in the horizontal and vertical boxes) for the KI67+ (left), KI67- (middle) populations (right: merge). Panel **(C)**: Total Intensity of the detected foci (calculated as sum of the intensity value of the pixels contained in all the spot masks in a single cell) well summarizes the amount of DNA damage including both nuclear diffused and foci enriched cells in the KI67+ (left), KI67- (middle) populations (right: merge).

Measurement of the γH2A.X signal at the lowest magnification and NA only identify high intensity stained cells, while foci was hardly detectable with the exception of large isolated spots in some nuclei (**Supplementary Figure 3**, **Figure 4**).

The developed image-cytometry analysis tools provided a precise classification of the DDR related phenotypes allowing counting and intensity measurement of the DD foci in the segmented cells. **Figure 4** reports the data measured on one representative core: number and intensity of foci in the epithelial areas were identified and subdivided according to proliferative activity of the targeted cells.

Different degrees of genomic damage were isolated according to the segmentation of γH2A.X foci and measurement of their area and intensity. The detection of a continuous range of signal spots allowed the isolation of γH2A.X highly expressing cells showing a signal diffused throughout the entire nucleus, and paradoxically exhibiting low number of foci. Mean intensity per nucleus and number and intensity of foci did not correlate at all as evidenced by the two distributions along the axis in **Figure 4** Panel B. The integrated Intensity of γH2A.X foci (**Figure 4** Panel C) thus represents a better indicator of the activated DDR response (42) by summarizing all the conditions associated to a highly damaged DNA i.e. few foci with high intensity versus numerous spots with lower mean intensity per object.

Classification of the damage in relation with the proliferative index underlined the link with replication stress. Proliferating cells show in the analyzed core (**Figure 4**, Panel A graph) higher values in all the DDR related parameters.

Analysis of 53BP1 content revealed a quite low expression level and no significant differences among proliferating and quiescent cells. Besides considering the γH2A.X foci as intracellular objects assigned to a targeted cell, the developed software produced statistics of the spots as an independent population not referred to any cell owner. Plot of the 53BP1 intensity per every γH2A.X spot (**Supplementary Figure 4**) revealed that high intensity foci of γH2A.X can (γH2A.X+53BP1+) or cannot (γH2A.X+53BP1-) show an elevated 53BP1 content. However, visual inspection of γH2A.X-53BP1+ spots revealed that a dimmed phosphorylated signal is frequently associated with antibody precipitates in the 53BP1 channel. At the end, a proper evaluation of the DNA Damage amount in the sample can be fully recapitulated by the analysis of γH2A.X only.

High-resolution acquisition of images is almost mandatory when profiling the transcriptional activity of cells by smRNA FISH. A combined immune-multiplexed/FISH experiment involves multiple steps in sample preparation and/or data acquisition that cannot be easily automated. smRNA FISH preparation protocols often require treatments that are not compatible with immune-detection of antigens (e.g. Proteinase K incubation). A sequential processing and data collection of the tasks related to the immune-detection and then in-situ hybridization can thus allow the completion of the entire experiment.

We adapted our sequential immunostaining protocol to perform combined protein and RNA detection. The samples stained according to the conditions listed above (i.e. KI67, CK8, γH2A.X, 53BP1) have been hybridized to detect the B-RAF and the β-Actin (ACTB1) transcripts with single molecule sensitivity. CK8 staining, initially acquired at the lowest resolution to select epithelium enriched cores, was employed to delimit cell borders for calculating the number of RNA spots per cell. KI67 expression was then used to define the proliferative index in the epithelial cells population.

To validate the efficiency of the stripping protocol with the maximum-sensitivity detection provided by the high NA objectives and to exclude potential artifacts by not removed antibodies, we evaluated the residual fluorescence detected in cells stained for the KI67, γH2A.X and 53BP1 nuclear antigens: no significant fluorescence residual was detected even in these conditions (**Supplementary Figure 5**).

**Figure 5:**
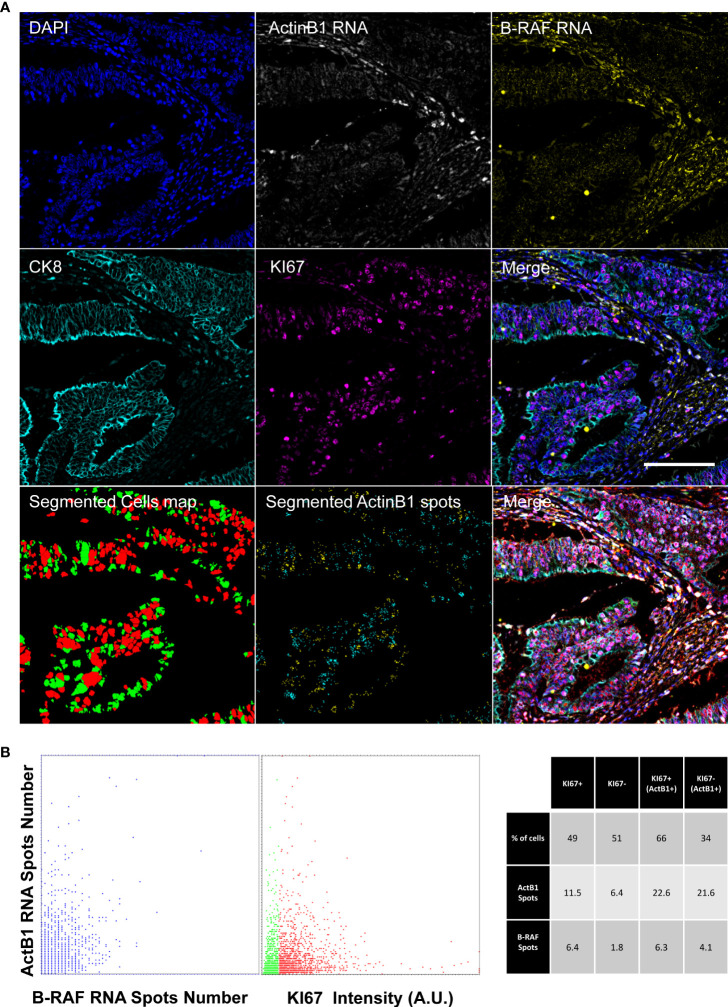
Diffraction-limited Image-Cytometry Analyis of a smRNA-FISH In-Situ hybridization. Images are collected with a 60x 1.4 Oil Immersion Objective to measure probe signals at maximum spatial resolution and sensitivity on a selected region inside a previously acquired core. Panel **(A)**: Images of DAPI, ActinB1 RNA, B_RAF RNA, CK8, KI67 (CK8, KI67 rescaled to the same digital resolution from the previously acquired 20x acquisition employed to target the selected ROI; scale bar: 200μm) and the resulting Merge are shown together with the masks of the segmented cells (KI67+, Red; Ki67- Green) and of ActinB1 spots of the isolated cell populations (KI67+ Cyan spots; KI67- Yellow spots). Panel **(B)**: The Dot Plot on the left reports the number of detected RNA spots per cell: the correlated distribution shows comparable hybridization efficiency for the two probes. In the Dot Plot the detected ActinB1 RNA spots number per cell are classified according to the KI67 content of the cell of origin(Red: KI67+; Green: KI67-). The table on the left shows a correlated analysis of KI67 content in relation to transcriptional activity (summarized by RNA spots number). Higher transcriptional acitivity (ActB1+), identified by an arbitrary threshold (>10) on ActinB1 spots evidences a link with the proliferating index. The proliferating fraction in this population is enriched (66% versus 49%) with respect to the entire cell population.

**Figure 5** shows the registered final images of the chosen representative core. ACTB1 was selected as measure of the general transcription level. The number of detected B-RAF foci showed a good correlation with the ACTB1 detected signal providing a read-out of the hybridization efficiency of the two RNA probes. Transcriptional activity was then correlated to the proliferative index: no major differences were observed among actively proliferating and quiescent cells. However, to exclude the influence of the potentially heterogeneous hybridization efficiency we restricted the analysis to the high content of ACTB1 RNA (a threshold was arbitrary set to 10 spots). Cells enriched in transcript exhibited a different repartition of the proliferating fraction among the population with a marked correlation among RNA content and KI67 expression.

We selected five additional cores with the highest fraction of epithelial tissue and analyzed for the same parameters (**Supplementary Figure 6**). The measured distributions showed high heterogeneity related to the biology of individual samples and also influenced by the variable efficiency in the preparation, staining and hybridization procedures detected from core to core (e.g. Signal-to-Noise Ratio greatly varied due the different autofluorescent levels; average number of ACTB1 FISH spots per cell varied from 1.2 to 8.9). The presented results thus provide an example of a high content analysis that can originate from the pipeline that can be considered a starting point for further clinically oriented studies. However, a validation of the biological data should require a focused approach with the selection and retrieval of samples presenting the phenotype of interest and analysis on more extended tissue slices with the appropriate replicas and statistical sampling.

### 3.3 Application of multiplexed pipeline with multimodal microscopy image collection: Combined three-dimensional immuno-multiplexed smRNA FISH analysis

A more reliable evaluation of the global amount of transcripts per cell can only be obtained by optical sectioning to allow reconstruction of the whole-cell volume. The same considerations could be extended to every cell feature requiring highly-resolved spatial analysis. Since high NA (i.e. maximum spatial-resolution) implies limited depth of focus, a complete description of such phenotypes requires the acquisition of multiple planes along the optical axis.

Even if scanners performing Z-stack reconstruction exist, a confocal microscope remains the best tool to couple high-resolution imaging to three-dimensional analysis. An automated acquisition driven by image-analysis removes the limits in its traditional low throughput. The developed computational tools allows single cells re-localization by calculating their positions in each picture and converting the image-coordinates into microscope stage-coordinates, thanks to the metadata stored in the acquired images. As a result, ROIs can be defined to target cells according to a specific phenotype. At each step slide position is automatically re-calculated by registering the low resolution maps, thus allowing acquisition at maximum resolution and bypassing the need of maintaining the sample fixed on the microscope. This way, only selected cores have been collected, avoiding re-acquisition of the entire TMA. This analysis-guided procedure allowed saving a huge amount of time employed in storing data that would be only in part useful for the final analysis.

As an additional feature, the high-resolution image-registration procedure generated multiplexed 3D data by acquiring and aligning Z-stacks of sequential stainings.

**Figure 6:**
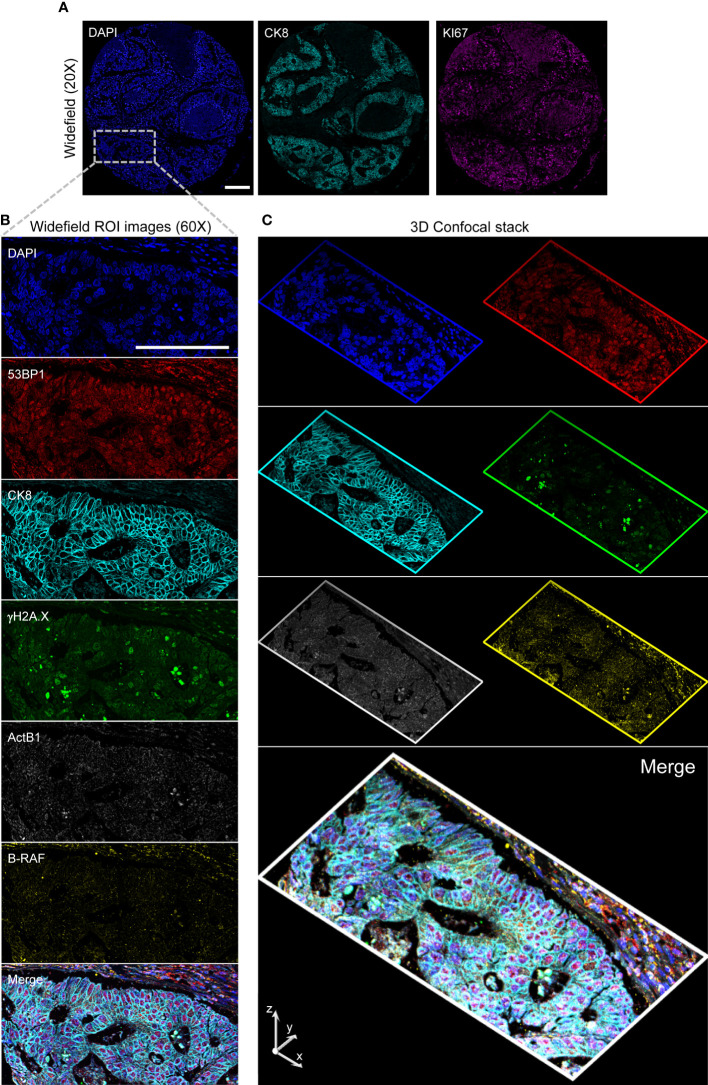
High-Resolution 3D Analysis of a targeted core. Panel **(A)**: Single cell resolved images (20x) of a selected core are collected and analyzed to target the CK8+ selected region (Inset. Blue: DAPI; Cyan: Ck8; Magenta: KI67) by conversion of the image- to microscope stage- coordinates. A 60x 1.4 NA Oil Immersion objective is then employed to acquire diffraction-limited widefield (Panel **B**) images and confocal stacks (Panel **(C)** stack max projections along the indicated axis) on the restained slide in two consecutive rounds. Images are then spatially registered to create the final result: Blue: DAPI; Red: 53BP1; Cyan: Ck8 (restained after stripping); Green: γH2A.X; Grays: ActB1 RNA FISH; Yellow: B-RAF RNA FISH. Scale Bar: 200μm.

To validate this multimodal pipeline, the TMA stained according to the scheme delineated in the previous paragraph was employed (**Figure 6**). After the above described low resolution acquisition, the cores have been first acquired at single cell resolution to measure KI67 and CK8 expression. Slides were then stripped, re-stained, and images collected at high resolution storing the signal for CK8, γH2A.X and 53BP1 plus DNA. Z-stacks of the different channels have then been acquired in specific user-defined areas in the selected tumor-enriched cores (a representative stack is included in the **Supplementary Material**). After another stripping step, the TMA was hybridized for RNA transcripts detection. The previously acquired cores were automatically located according to the above described procedure. High resolution widefield images and confocal stacks were thus collected and then finely registered to get the final 3D stacks.

As a final result, specific regions were targeted providing a planar diffraction-limited resolution description of DDR and smRNA FISH with 6 parameter confocal stacks generated after 3D spatial alignment (a representative stack is included in the **Supplementary Material**: s18_Hyperstack_c=6_z=13).

## 4 Discussion

Spatial Biology revolutionized the approach to cancer comprehension by introducing over the framework of Next Generation Sequence (NGS) the mapping of the network of cellular and molecular interactions taking place between the tumor and the surrounding microenvironment.

High content analysis is present in numerous fields since years: NGS, proteomics, flow cytometry are all tools available in laboratories to generate and analyze an enormous amount of data. However, NGS technologies base their action on the averaging assumption, i.e. loose of the cellular resolution in a population. Unfortunately, almost all the high content mentioned tools are not able to include a spatial description of the constituents of the sample.

Imaging and microscopy can provide a well detailed picture but are frequently limited in their throughput. In the last years, instrument automation, protocol developments and high end image analysis based on deep learning transformed them making one of the best tools for a Multiplexed experimental approach.

However, executing a multiplexing high-content experiment is nowadays a challenge requiring expensive instrumentation and technological skills.

We presented here a novel pipeline to perform in-situ spatial proteomic analysis based on the automation of all the phases of the process. The resulting workflow aims at reducing costs and times, simultaneously maintaining high quality data, by employing not dedicated instrumentation that is usually present in the vast majorities of the laboratories.

The work was focused on the optimization of every single step in the process:

- sample staining, alternating very high speed thanks to automated staining, and high efficiency staining at the bench;
- data collection, with automation of a fluorescence microscopy workstation guided by parallel image analysis;
- data analysis, by providing a series of open-source image cytometry tools.

The fluorescence microscope is able to reach a spatial resolution ranging from single cells to single molecules. Huge histological samples can be efficiently analyzed by the traditional widefield microscope, merging single cell resolution, speed and sensitivity. Spatial resolution can be pushed to the diffraction limit opening a window on a detailed description of the intracellular space and of the molecular interactions taking place therein. The concept of analysis-driven acquisition consists in choosing the right image collection conditions for the biological task addressed by the current experimental question. This step-by-step image collection with increasing resolution allows i) optimizing the time of acquisition and data storage (and consequently subsequent analysis) by saving only informative data, thanks to the ROI-limited data collection; ii) maximizing the spatial resolution and sensitivity thanks to the employment of high NA oil-immersion objectives.

For their structure, TMAs constitutes one of the best demonstrations to validate the approach. Online re-localization of structures, by converting the image coordinates to microscope stage positions, allows starting from the collection and analysis of cores considered as the targeted spatial entity. Time and storage resources were optimized limiting the acquisition to their real spatial extension.

Even if fluorescence scanners provide an easy to use solution to automate image acquisition, their major limitation is an observation limited to “dry” objectives. The consequent exclusion of high Numerical Aperture objectives precludes the investigation of diffraction-limited details and lowers the sensitivity. However, coverage of large areas can be hampered by the requested storage resources. A data collection restricted to significant regions is thus mandatory to overcome the problem.

The analysis of high resolution details in pathology samples is a growing demand that is stimulating new approaches (43). Besides the possibility to facilitate execution of diagnostic assays by employing optical microscopy in place of electron microscopy when needed, even more routine examinations can benefit of high spatially resolved information. Single cell segmentation performances can be greatly improved by highly contrasted and resolved imaging thus completing the classical analysis performed by pathologists with a more objective fully-automated approach.

Diffraction-limited observation can introduce novel phenotypes for the comprehension of the molecular mechanisms regulating cell growth that can be altered in transformation. We provided analysis of DNA Damage related foci as proof-of-principle. Low resolution observations can influence the correct interpretation of results by providing a view limited in resolution and sensitivity. Uniform γH2A.X high intensity staining, which was detected in images with cell resolution (20x) (**Supplementary Figures 3, 4**), correlates with lethality induced by replication stress (44). However, diffraction-limited analysis revealed a huge number of Double Strand Breaks by detecting γH2A.X foci. They can be associated to high genomic instability originated by replication stress that favors the onset of mutations, thus driving new clones evolution. The causal link with the replicating activity can be further reinforced by an analysis correlated to the proliferative index: proliferating cells showed more than doubled levels of histone phosphorylation with respect to their quiescent counterpart in the analyzed epithelium. All this features cannot be extrapolated by a standard cell-resolved analysis that is unable, as demonstrated, to detect the finest intracellular spatial details.

Spatial Transcriptomics is rapidly evolving towards in-situ detection of thousands of genes. Even if the developed amplification techniques (21, 22) greatly enhances signals from single RNA molecules making it detectable even with medium sensitivity and resolution, high-resolution optical microscopy provides a signal-to-noise ratio that reduces error in the correct detection, localization and assignment of the RNA spots. Sequential acquisition-analysis rounds allowed the correlation of single-cell profiling to transcriptional activity to underline links among different cellular processes. In some targeted cores of the analyzed sample, both DNA transcription and replication associated to high levels of proliferation and consequently to DNA Damage thus remarking a possible dual origin for the observed genomic instability (45).

Moreover, the employed variable resolution workflow naturally introduces a “Multi-Modal Microscopy” approach allowing the switching between different imaging modalities. Spatial resolution in modern fluorescence microscopy can be greatly improved by a plethora of sister technologies (e.g. Confocal Microscopy, Spinning Disk Confocal Microscopy, Lightsheet Microscopy, Super-Resolution by Single-Molecule Localization Microscopy, Stimulated Emission Depleted Microscopy) built around the optical fluorescence microscope.

A sequential pipeline, allowing the step-by-step adaptation of sample preparation, observation and analysis, opens the possibility i) to modulate the acquisition parameters to address a specific question, e.g. moving from cell resolved to intracellular diffraction-limited analysis; ii) to modify the employed optical technology, e.g. providing a 3D analysis by confocal microscopy. We demonstrated that such a workflow can be efficiently used to re-locate areas of interest to be imaged and that registration of different acquisitions, even in 3D, can be an efficient alternative way to increase the number of parameters and consequently the content of a multiplexed analysis.

Employment of high resolution imaging in multiplexing experiments is contrasted by the need of a restricted field of view at increasing resolution: an apparently unresolvable contradiction in the histological framework where observation is extended instead to large tissue areas. The approach we presented provides a possible compromise by restricting the request of augmented resolution to spatially confined regions. A previous analysis step for identification and localization of a phenotype of interest allows an intelligent selection of the targets, instead of a random or human-driven selection. Extension to the entire sample and simultaneously provides statistical significance and optimization of the storage and computational resources.

## 5 Concluding remarks

The heterogeneous and complex nature of cancer constitutes an enormous challenge on the walk towards disease cure and eradication. More and more evidences suggest that its comprehension requires analysis of the network of interactions established among cancer cells inside the tumor and of the onset of communications channels with the host environment surrounding it.

However, technological evolution frequently focuses on specific tasks to facilitate diffusion of the developed solutions. The enormous diversity among samples and the deriving plethora of biological questions to be addressed suggest instead that, at least in specific situations, a high degree of flexibility has to be preserved.

An alternative choice leads instead to merge already developed solutions that efficiently answer selected questions. Optical microscopy has *per se* a heterogeneous nature that offers high flexibility with the advantage of a long-time established use.

Since years, cancer research has been developing different experimental models to replicate in the laboratory cancer-onset and development: tissue biopsies, in-vitro patient-derived organoids, animal models including patient-derived xenografts. Personalized-medicine benefits of the discoveries from each of these fields. A class of technological solutions able to dynamically adapt themselves to this diversity, maintaining simultaneously the same conceptual framework, can be consequently extremely advantageous. Automation can be the first step in making traditional instrumentation, and in particular optical microscopy, able to operate in the multi-omics environment. Second, Artificial Intelligence can favor the dissemination of solutions employing an already familiar series of technological tools, i.e. fluorescence microscopy, by introducing novel analysis routines in the field of histopathology, where a higher degree of objectivity is required to lighten the workload still almost completely based on human examination.

## Data availability statement

The raw data supporting the conclusions of this article will be made available by the authors, without undue reservation.

## Ethics statement

The studies involving human participants were reviewed and approved by Institutional Review Board Comitato Etico Brianza, N. 3204, “High-dimensional single cell classification of pathology (HDSSCP)”, October 2019, San Gerardo Hospital in Monza. The patients/participants provided their written informed consent to participate in this study.

## Author contributions

All authors contributed to the conceptualization and design of the pipeline and participated to the writing of the manuscript. LF, SP and FP carried out the experiments. SP and MF produced the software tools and carried out data analysis. MB and GC implemented and supervised the multiplexing protocol. PP contributed to retrieve funding for the realization of the work. MF designed and supervised the experiments, and wrote the manuscript. All authors contributed to the article and approved the submitted version.

## Funding

This work was partially supported by the Italian Ministry of Health with Ricerca Corrente and 5x1000 funds.

## Acknowledgments

MF wants to thank Nicolas Duthilleul for the technical support and thoughtful inputs with the automated stainer.

## Conflict of interest

The authors declare that the research was conducted in the absence of any commercial or financial relationships that could be construed as a potential conflict of interest.

## Publisher’s note

All claims expressed in this article are solely those of the authors and do not necessarily represent those of their affiliated organizations, or those of the publisher, the editors and the reviewers. Any product that may be evaluated in this article, or claim that may be made by its manufacturer, is not guaranteed or endorsed by the publisher.
