# Supplementary material for: Automated multimodal fluorescence microscopy for hyperplex spatial-proteomics: Coupling microfluidic-based immunofluorescence to high resolution, high sensitivity, three-dimensional analysis of histological slides
Source: Front Oncol. 2022 Oct 13;12:960734. doi: 10.3389/fonc.2022.960734 (PMC9606676; doi:10.3389/fonc.2022.960734)
Supplement: Supplementary file 1 [file DataSheet_1.zip › Supplementary_Material16082022.docx]

Supplementary Material

# Supplementary Files

## s18_Hyperstack_c=6_z=13. Representative multichannel confocal stack of a relocalized area. Stack can be opened by ImageJ setting hyperstack dimension to 6 channels and 13 z planes. Channels order: 1. DAPI; 2. CK8; 3. 53BP1; 4. γH2A.X; 5. ACTB1 FISH; 6. B-RAF FISH.

## .s18_WF20X_AcqMetadata. CSV file containing the metadata of a 20x widefield representative acquisition.

## s18_WF60X_AcqMetadata. CSV file containing the metadata of a 60x widefield representative acquisition.

## s18_Conf_DDR_AcqMetadata. CSV file containing the metadata of a representative confocal acquisition of an experiment to detect DDR parameters.

## s18_Conf_FISH_AcqMetadata. CSV file containing the metadata of a representative confocal acquisition of a FISH experiment.

## multiplexingcenterTMA.bin. Bin file of the NIS Software procedure employed for the parallel acquisition and registration of images described in the paper. It can be opened and worked on Nikon systems with the JOB package only. Use of the procedure requires knowledge of the JOB software and must be adapted to the specific set up. The file is provided to work as a template for customization.

## JOB.png. Screenshot of the procedure in NIS, Nikon Acquisition Software.

## MacroRegTwoImagesforNIS_22072022. Source code for the ImageJ macro launched by NIS to perform first image alignment, correction of the shift and saving of the acquisition ROI then employed by the acquisition software NIS.

# Supplementary Table

## Supplementary Table 1. List of the antibodies used in the automated staining with the employed dilutions.

## Supplementary Table 2. Comparison of the time requested by the manual *versus* automated sample-preparation steps.

# Supplementary Figures

**Supplementary Figure 1.** Multiplexed automated staining of different antigens by a microfluidic stainer on tonsil tissue section. Antibodies list is reported in Supplementary Table 2. Scale Bar: 200 μm.

**Supplementary Figure 2.** Characterization of the staining performances of a microfluidic based automated stainer. Primary antibodies incubation time were tested in comparison to an overnight incubation at the bench. Incubation time of 4 min (upper row) with a microfluidic stainer versus overnight at the bench (lower row) for 𝛾H2A.X, 53BP1 and Histone H3 nuclear markers. Scale Bar: 50 μm.

**Supplementary Figure 3.** Efficiency of detection of γH2A.X foci in standard, cell resolved conditions with a 20x magnification 0.7 NA dry objective (left) in comparison to a diffraction-limited observation with a 60x 1.4 NA oil-immersion objective. Scale Bar: 100 μm.

**Supplementary Figure 4.** Distribution of the 53BP1 protein content in γH2A.X foci. The Dot Plot shows the intensity of the 53BP1 signal in comparison to the γH2A.X intensity in γH2A.X foci. The vast majority of events shows a correlation of the two signals (γH2A.X +53BP1+) with a small subpopulation of foci accumulating only the phosphorylated histone without accumulation of 53BP1 (γH2A.X +53BP1-). The poorly represented class of foci with the inverted distribution (γH2A.X low and 53BP1 high content; γH2A.X -53BP1+) coincides with spots generated by antibodies background as revealed by visual inspection.

**Supplementary Figure 5.** Immunofluorescence images of a representative core pre- and post- stripping procedure. Core was labelled with DAPI for DNA staining and with the following antibodies: γH2A.X (Alexa Fluor 647), KI67 (Alexa Fluor 555) and 53BP1 (Alexa Fluor 488). ROI images and histograms showed quantification of fluorescence intensity after staining and stripping in each channel. Scale bar: 200 𝜇m.

**Supplementary Figure 6.**  Analysis of DDR and transcriptional activity parameters on cores selected for the highest enrichment in the epithelial fraction. Cytokeratin 8 negative portions were not included in the analysis employing the analysis on 20x acquired images reported in the article. Statistics have been calculated on areas of linear extension of at least 500 microns. The horizontal axis reports a code identifying the position of the cores in the TMA. The vertical axis reports the ratio of the values for the defined parameter (shown in the title of graph) measured in the KI67 negative and positive cell populations.
