# Supplementary material for: Automated multimodal fluorescence microscopy for hyperplex spatial-proteomics: Coupling microfluidic-based immunofluorescence to high resolution, high sensitivity, three-dimensional analysis of histological slides
Source: Front Oncol. 2022 Oct 13;12:960734. doi: 10.3389/fonc.2022.960734 (PMC9606676; doi:10.3389/fonc.2022.960734)
Supplement: Supplementary file 1 [file DataSheet_1.zip › SuppTable1.docx]

| **Name** | **Isotype** | **Cat n.** | **Source** | **Concentration** |
| --- | --- | --- | --- | --- |
| Anti-CD4 | Rabbit | ab133616 | Abcam | 1,66 µg/ml |
| Anti-CD45ro | Mouse IgG2A | [sc-1183](https://www.scbt.com/scbt/product/cd8-alpha-antibody-c8-144b?productCanUrl=cd8-alpha-antibody-c8-144b&_requestid=937902) | SCBT | 2 µg/ml |
| Anti-CD20 | Mouse IgG2A | sc-58985 | SCBT | 2 µg/ml |
| Anti-CD68 | Mouse IgG1 | sc-20060 | SCBT | 2 µg/ml |
| Anti-CD16 | Mouse IgG1 | sc-20052 | SCBT | 2 µg/ml |
| Anti-HLA-DR | Mouse IgG2b | sc-56545 | SCBT | 2 µg/ml |
| Anti PD-1 | Goat | AF1086 | R&D system | 5 µg/ml |
| CTLA4 | Mouse IgG1 | UM800141 | Origene | 5 µg/ml |
| Anti p53 | Mouse IgG2a | sc-126 | SCBT | 1 µg/m**l** |
| γH2A.X | Mouse IgG1 | 613402 | Biolegend | 5 µg/ml |
| 53BP1 | Rabbit | 36823 | Abcam | 5 µg/ml |
| Anti PD-L1 | Rabbit | ab213524 | Abcam | 4,96 µg/ml |
| Anti- Ki67 | Mouse IgG1 | 550609 | BD | 5 µg/ml |
| Anti-Histone cluster 1 H3D | Mouse IgG3 | sc-134355 | SCBT | 2 µg/ml |
