# Supplementary material for: Automated multimodal fluorescence microscopy for hyperplex spatial-proteomics: Coupling microfluidic-based immunofluorescence to high resolution, high sensitivity, three-dimensional analysis of histological slides
Source: Front Oncol. 2022 Oct 13;12:960734. doi: 10.3389/fonc.2022.960734 (PMC9606676; doi:10.3389/fonc.2022.960734)
Supplement: Supplementary file 1 [file DataSheet_1.zip › SuppTable2.docx]

| MILAN protocol | Bench | LabSat |
| --- | --- | --- |
| Dewaxing | 30 min | At the bench |
| Antigen Retrieval | 30 min + cooling time (15 min) | 20 min |
| Primary Antibodies | Overnight (18 h) | 4 min  10 + 10 min |
| Secondary Antibodies | 1 h | 4 min |
| Mounting and Image Collection | from 1 to several hours according to the experiment | Identical |
| Stripping | 1 h | At the bench |
| Restaining | Overnight + 1h minimun | 30 min |
|  |  |  |
| Total | About 20 hours for the first passage; 1 h + 20 hours at each restaining step | About less than 2 hours and 1h + 0.5 hours at each restaining step |
