# Supplementary figures and images for: Automated multimodal fluorescence microscopy for hyperplex spatial-proteomics: Coupling microfluidic-based immunofluorescence to high resolution, high sensitivity, three-dimensional analysis of histological slides
Source: Front Oncol. 2022 Oct 13;12:960734. doi: 10.3389/fonc.2022.960734 (PMC9606676; doi:10.3389/fonc.2022.960734)

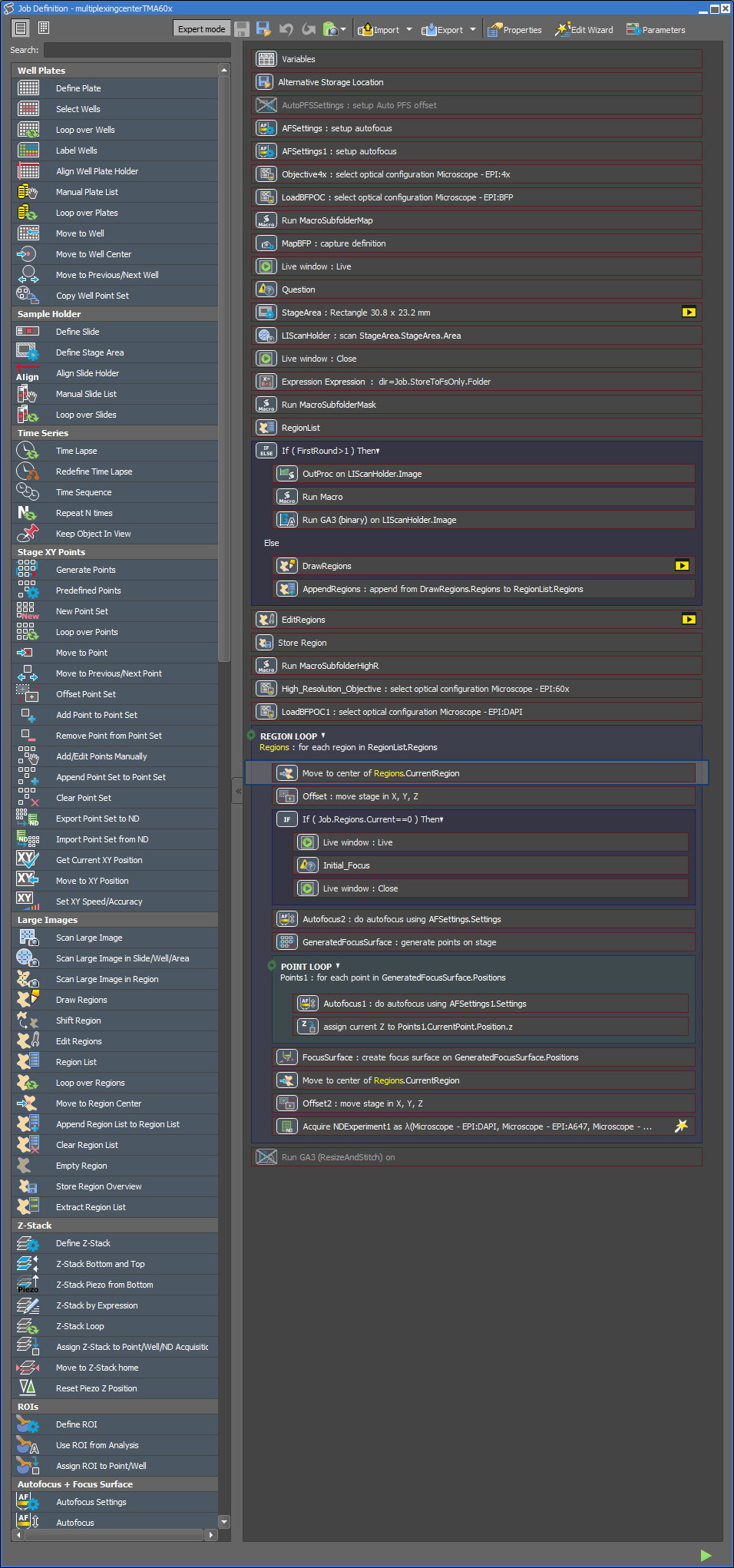

Supplement: Supplementary file 1 [file DataSheet_1.zip › JOB.PNG]

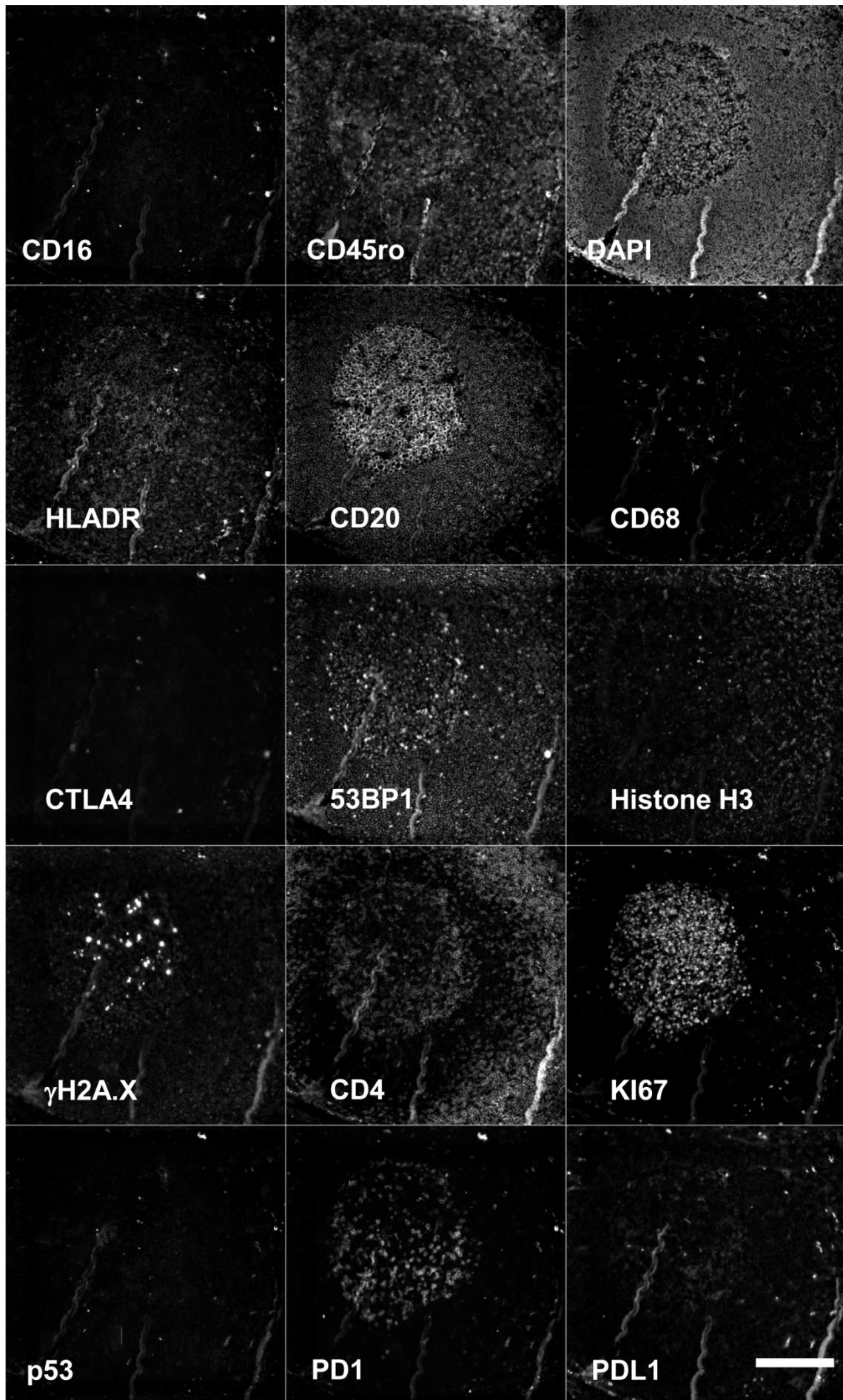

Supplement: Supplementary file 1 [file DataSheet_1.zip › SupplementaryFigures/SuppFig1.pdf]

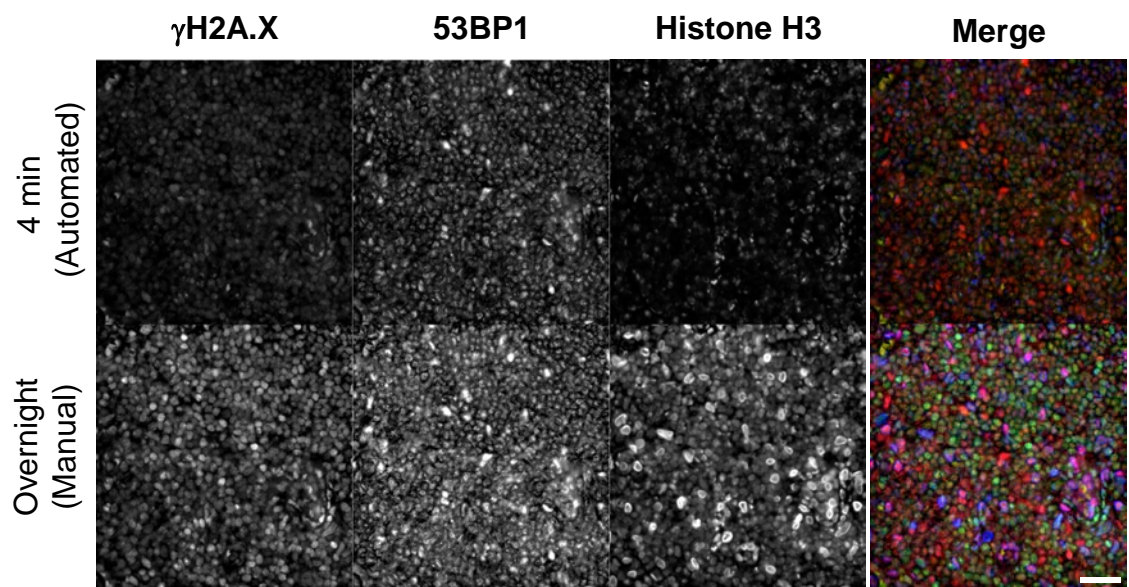

Supplement: Supplementary file 1 [file DataSheet_1.zip › SupplementaryFigures/SuppFig2.pdf]

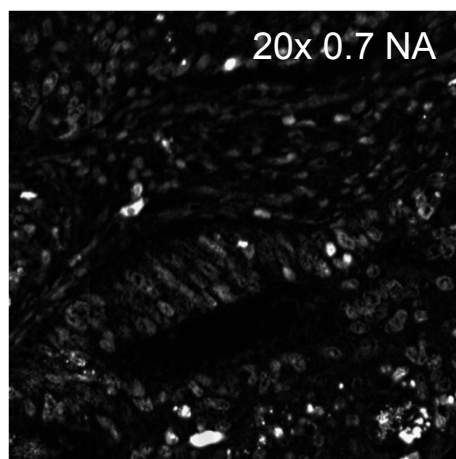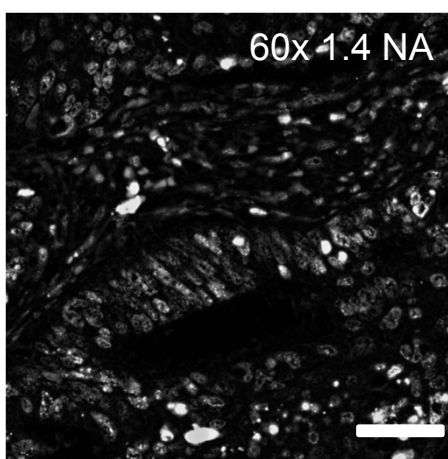

Supplement: Supplementary file 1 [file DataSheet_1.zip › SupplementaryFigures/SuppFig3.pdf]

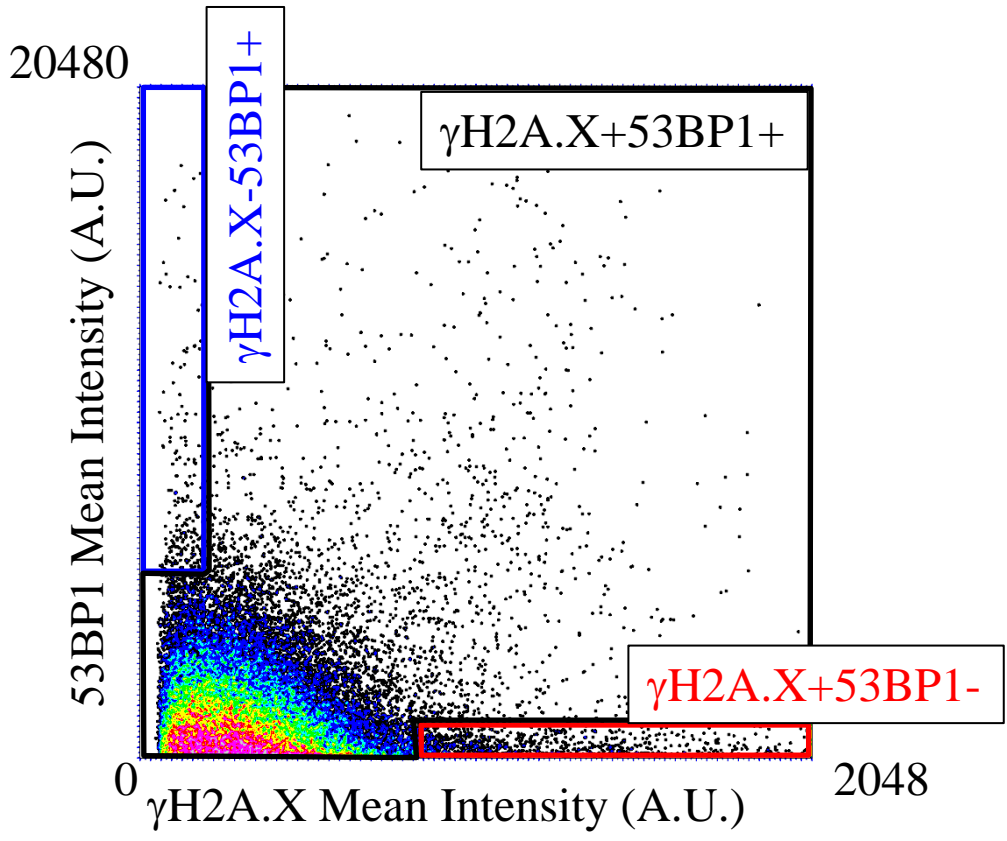

Supplement: Supplementary file 1 [file DataSheet_1.zip › SupplementaryFigures/SuppFig4.pdf]

Staining

Stripping

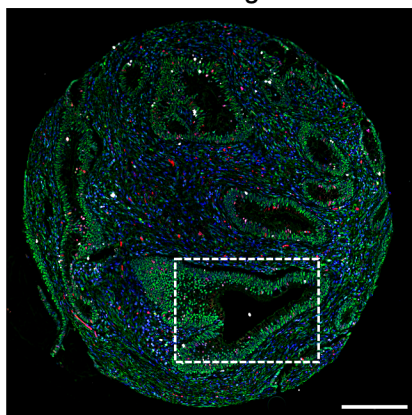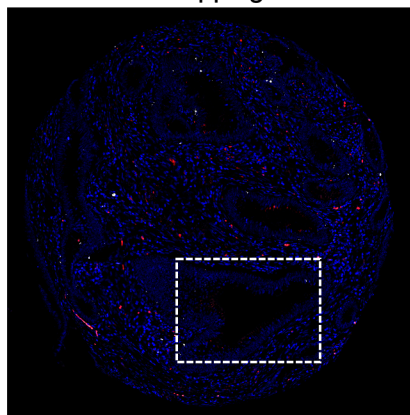

Pre-Stripping

Post-Stripping

DAPI

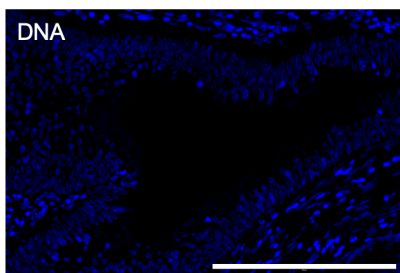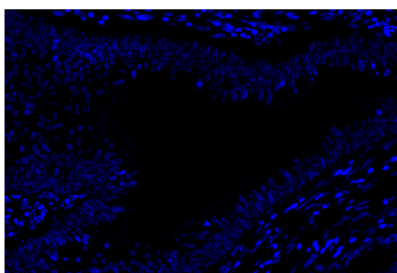

Alexa Fluor 647

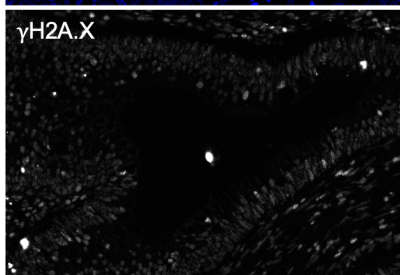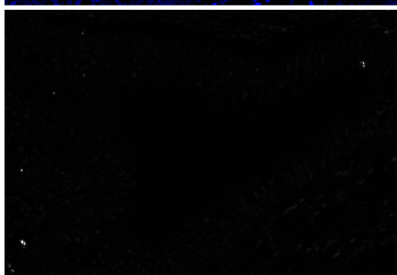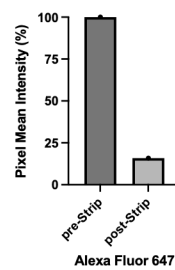

Alexa Fluor 555

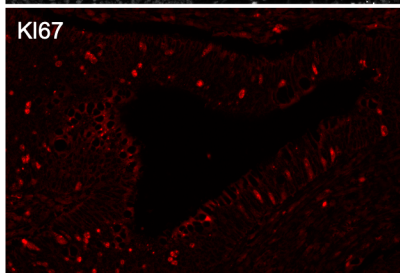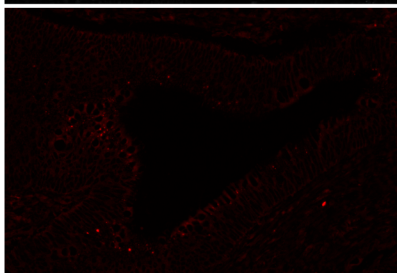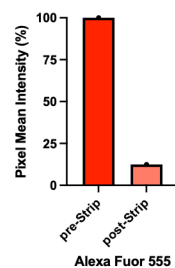

Alexa Fluor 488

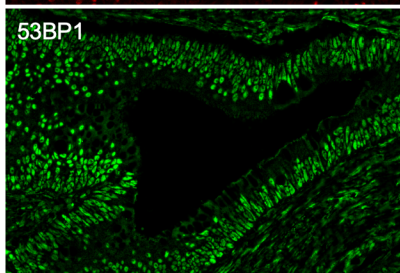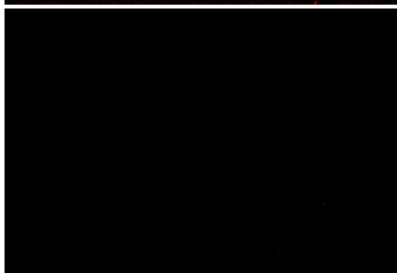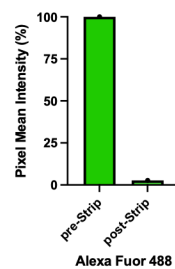

Supplement: Supplementary file 1 [file DataSheet_1.zip › SupplementaryFigures/SuppFig5.pdf]

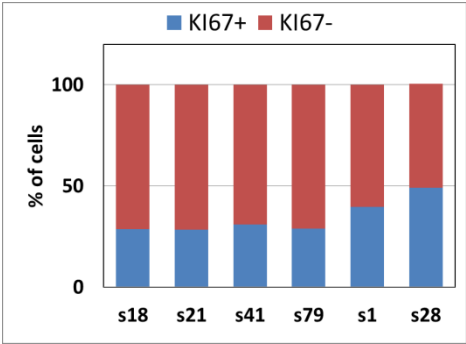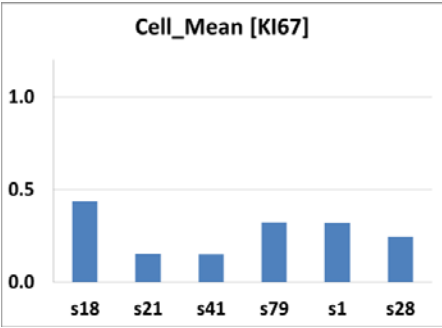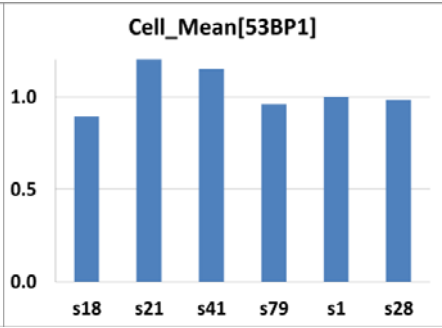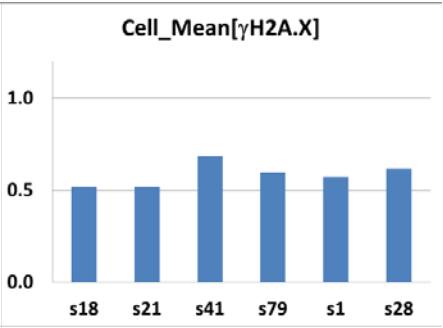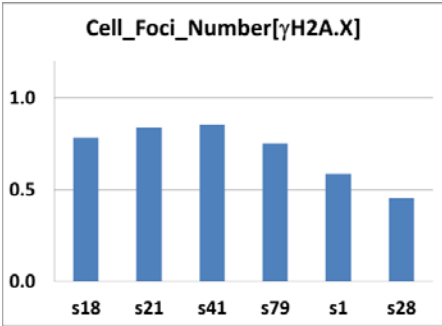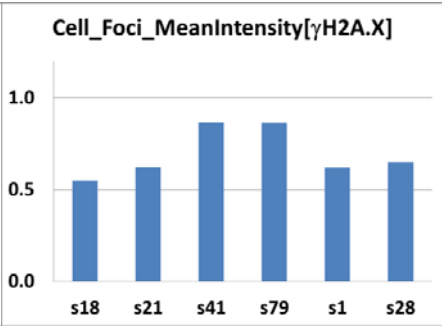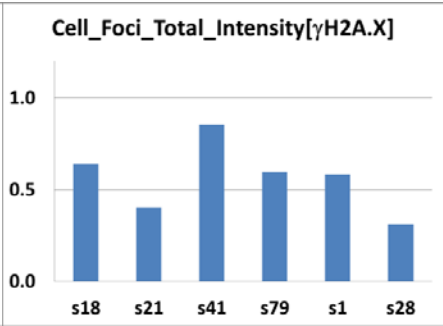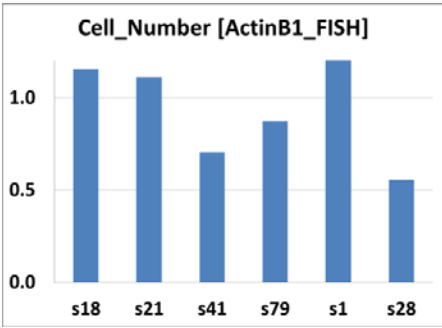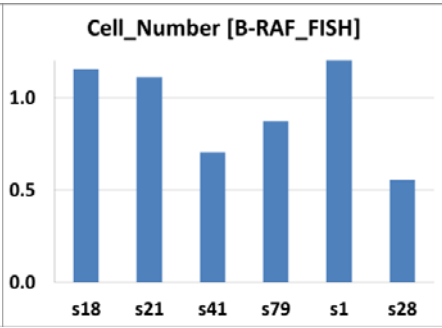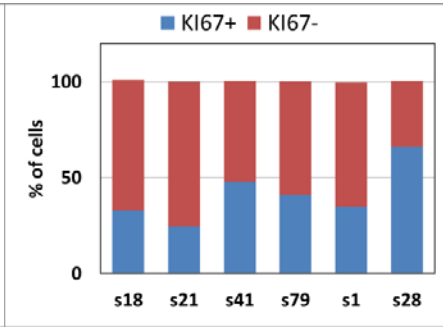

KI67- / KI67+ Ratio

Core Identificative Code

Supplement: Supplementary file 1 [file DataSheet_1.zip › SupplementaryFigures/SuppFig6.pdf]
